# Ets-2 Regulates Cell Apoptosis via the Akt Pathway, through the Regulation of Urothelial Cancer Associated 1, a Long Non-Coding RNA, in Bladder Cancer Cells

**DOI:** 10.1371/journal.pone.0073920

**Published:** 2013-09-12

**Authors:** Wenjing Wu, Shuwan Zhang, Xu Li, Mei Xue, Sancheng Cao, Wei Chen

**Affiliations:** 1 Clinical Laboratory, the First Affiliated Hospital, School of Medicine, Xi’an Jiaotong University, Xi’an, China; 2 Translational Medicine Center, the First Affiliated Hospital, School of Medicine, Xi’an Jiaotong University, Xi’an, China; 3 Clinical Laboratory, Xi’an Children’s Hospital, Xi’an, China; UCSF/VA Medical Center, United States of America

## Abstract

The majority of the human genome is transcribed and generates non-coding RNAs (ncRNAs) that fail to encode protein information. Long non-coding RNAs (lncRNAs) are emerging as a novel class of ncRNAs, but our knowledge about these ncRNAs is limited. Previously, our laboratory has identified that a lncRNA, Urothelial cancer associated 1 (UCA1), played an important role in bladder cancer. Despite the recent interest in UCA1 as a diagnostic marker for bladder cancer, little is known about its transcriptional regulation. To elucidate the regulation of UCA1 gene expression, we have characterized the human UCA1 gene promoter. A 2.0-kb fragment of its 5′ flanking region was cloned into a luciferase reporter vector. Deletion and mutation analysis suggested that an Ets-2 binding site was critical for UCA1 gene promoter activity. Further analysis of this site by gel shifting, chromatin immune precipitation (ChIP), and co-transfection experiments showed that transcription factor Ets-2 directly bound to the UCA1 promoter region and stimulated UCA1 promoter activity in bladder cancer cells. Taking into account the anti-apoptosis function of Ets-2, our data suggested that Ets-2 regulates apoptosis process by regulating the expression of UCA1, moreover UCA1 may be involved in the activation of Akt signaling pathway by Ets-2 in bladder cancer cells.

## Introduction

Eukaryotic genomes are not the simple, well-order substrates of gene transcription that was once believed. We now know them to transcribe a broad spectrum of RNA molecules, ranging from long protein-coding mRNAs to short non-coding transcripts, which frequently overlap or are interleaved on either strand [1]. Long non-coding RNAs (lncRNAs) are emerging as a novel class of non-coding RNAs containing more than 200 nucleotides, but our knowledge of these lncRNAs is limited. LncRNAs are the transcripts that resemble protein-coding mRNAs in that they are capped, spliced and polyadenylated RNA polymerase II transcripts. They differ from mRNAs only in their lack of protein-coding open reading frames (ORF) [2].

In contrast to the diversity of RNA species, only a small number of lncRNAs were identified to have experimentally-derived functions. Moreover, the dysregulation of lncRNA has been shown in various types of cancer [3], [4], such as MALAT-1 in lung cancer [5], HULC in hepatocellular carcinoma [6], and HOTAIR in breast cancer [7], indicating that lncRNAs may play a critical role in tumorigenesis or tumor progression.

Previously, we established BLZ-211 and BLS-211 cells which are a pair of bladder transitional cell carcinoma (TCC) cell lines cloned separately from the same patient’s sample, but with different biological characteristics [8], [9], [10]. Then We reported a novel expressed sequence tag (EST) (Genbank accession number DR159656) isolated from these two cell lines by using subtractive suppression hybridization (SSH) technique [11]. Based on this EST, we cloned and identified a ncRNA named Urothelial cancer associated 1 (UCA1) (Genbank accession number EU334869) [12]. UCA1 belongs to the lncRNAs due to the lack of a strong Kozak consensus sequence for translation initiation in any of the ATG codons at multiple small ORFs [12]. Many studies suggested that UCA1 may act as a molecular marker of bladder cancer because of its excellent specificity and sensibility [13], [14]. A previous study in our laboratory also implied that elevated UCA1 expression can influence bladder cancer cell growth and promote invasion of the bladder cancer cells [12], suggesting it would be a novel therapeutic target gene of bladder cancer. Despite this interest, little is known about the *cis*-regulatory elements directly involved in the transcriptional modulation of the UCA1 gene in bladder cancer. In addition, the study by our laboratory showed that there are three splicing variants of the UCA1 gene existing in bladder cancer cells [12], [15]. The elevated expression of UCA1 in bladder cancer and the existence of splicing variants strongly indicate that the transcriptional regulation of the UCA1 gene is tightly controlled. The UCA1 *cis* regulation may shape complex gene expression networks that ultimately drive biological processes, such as cell apoptosis.

In this study, we identified the promoter of the human UCA1 gene for the first time and investigated the regulation of the UCA1 promoter by the Ets-2 transcription factor. Ets-2 can influence bladder cancer cell apoptosis by regulating the expression of UCA1. We also found lncRNA UCA1 may be involved in the activation of the Akt pathway. This research will provide insight into the regulatory mechanism of UCA1 expression in further studies.

## Materials and Methods

### Promoter Prediction

Previously, the full length of the UCA1 cDNA and its transcriptional start site (TSS) were identified using SMART RACE technology [12]. In this study, MegaBLAST (NCBI) program was used to map the UCA1 gene and its 5′ flanking region and DNA sequences were downloaded from GenBank (NC_000019.9). The TSS of the UCA1 gene was marked as +1. To predict the promoter and putative transcription factors, the following some online software were utilized. The promoter and TSS predict tools include the FPROM program from Softberry software (http://linux1.softberry.com/berry.phtml?topic=fprom&group=programs&subgroup=promoter) and PromoterInspector program from genomatix (http://www.genomatix.de/online_help/help_gems/PromoterInspector_help.html) [16]. The MatInspector program from genomatix (http://www.genomatix.de/online_help/help_matinspector/matinspector_help.html) [17], [18] was used for the transcription factor prediction.

### Plasmid Constructs

To generate pGL3-UCA1-2000 plasmid, a DNA fragment containing the *cis* region of the UCA1 gene from −1800 to +200 bp was amplified by PCR (PrimeSTAR® HS DNA Polymerase, TaKaRa, Dalian). The PCR product was then excised from agarose gel and isolated by using the Agarose Gel DNA Fragment Recovery Kit (TaKaRa, Dalian). The purified DNA fragment was then inserted into pGL3-Basic luciferase expressing vector (Promega, USA) through *Kpn*I and *Nhe*I sites. Other truncated fragments were made in the above described way. The primer sets used for this series of products were provided in Table 1. The amplified DNA fragments were inserted into pGL3-basic vector through the same restriction endonucleases sites. All DNA constructs were sequenced.

**Table 1:** Primer sets used in the present study.

Target		Sequence
*For Luciferase assay*		
Universal reverse primer (underlined: *Nhe*I site)		5′-CTAGCTAGCTGTGTGAGCAACAAGGCTGTTAATT-3′
Forward primers(underlined: *Kpn*I site)		
pGL3-UCA1-2000	−1800–+200	5′-CGAGCTCTATTAATCTCTGAAGTCCAGGTACCAGG-3′
pGL3-UCA1-1200	−1000–+200	5′-CGAGCTCTGTAATCCCAGCACTTTGGGAGTT-3′
pGL3-UCA1-900	−700–+200	5′-CGAGCTCTTGCGTCACCTCAGTGAAGGTG-3′
pGL3-UCA1-600	−400−+200	5′-CGAGCTCTCTCAGGCTGTCCTCTGGGAAG-3′
pGL3-UCA1-350	−150–+200	5′-CGAGCTCGGAGCCAAGAAGTCTGGAGCAG-3′
*For site-directed mutagenesis*		
pGL3-UCA1-1200-mut	sense	5′-CCTCAGGCTGTCCTCTAAGCTTAAATGACCCAGGAGC-3′
	antisense	5′-GCTCCTGGGTCATTTAAGCTTAGAGGACAGCCTGAG-3′
*For ChIP*		
UCA1-Ets-2	sense	5′- CTTTTAGATGACGGAGGGAGATACCAG -3′
	antisense	5′- CTGCCTGGGGCTCATCTGAGAT -3′

### Site-directed Mutagenesis

Substitution mutation of Ets-2-binding sites was generated by the PCR-based site-directed mutagenesis (Byotime, China). The pGL3-UCA1-1200 vector was used as the DNA template, and the Ets-2 core binding site (GGGAAG) was replaced to a *Hind*III restriction site (AAGCTT) (Table 1). The mutant vector was named as pGL3-UCA1-1200-mut. The mutations were confirmed by restriction enzyme digestion and sequencing.

### Cell Culture, Transient Transfection

Human bladder transitional cell carcinoma (TCC) cell lines BLZ-211 and BLS-211 were cultured in RPMI 1640 medium (Gibco-BRL, Gaithersburg, MD, USA). BLZ-211 and BLS-211 cell lines were established by our lab in 1994. The bladder cancer tissue was from surgical specimens of a 55-years old man with the diagnosis of multifocal papillary transitional cell carcinoma (TCC) grade II. All the procedure was administrated unber the approval of ethics committee of the First Affiliated Hospital, School of Medicine, Xi’an Jiaotong University [8]. Cells with more than 80% confluence were used for transfection.

BLZ-211 cells were transfected with FuGENE®HD Transfection Reagent (Roche, USA) in 24-well plate. Each well contained 2×10^5^ cells, 0.5 µg of pGL3-UCA1 series vector, 0.02 µg of the internal control vector pRL-TK (Promega, USA), 1 µl FuGENE®HD, and 500 µl RPMI 1640 medium without serum or antibiotics. pGL3-basic (Promega, USA) vector was transfected as control. For siRNA transfection, cells were transfected with X-tremeGENE siRNA Transfection Reagent (Roche, USA) in 6-well plate. Both Ets-2 siRNA and scrambled control siRNA are commercial products (Santa Cruz, USA).

### Luciferase Assays

Prior to assay cells were rinsed with phosphate buffered saline (PBS) at 48 hours after transfection and lysed in a passive lysis buffer (Promega, USA). Luciferase activity was measured by a luminometer (Promega, USA) using the Dual-Luciferase reporter assay kit (Promega, USA). Result was normalized to the Renilla luciferase activity. Reported data were represented as the mean from three independent experiments.

### Electrophoretic Mobility Shift Assay (EMSA)

Nuclear proteins were extracted from the BLZ-211 cells using nuclear protein extract kit (Byotime, China). Then electrophoretic mobility shift assay was performed according to EMSA Kit (Pierce, USA). Then probes were prepared based on the Ets-2 binding site in the UCA1 gene promoter and its corresponding complementary sequence (5′-TGTCCTCTGGGAAGAAATGACC-3′) called UCA1-wt-Ets accompanied with a mutant version (5′-TGTCCTCTGTATAGAAATGACC-3′) as well, named UCA1-mut-Ets. A 5′-biotin modification was included in UCA1-wt-Ets probe. For competition assays, 200-fold excess of unlabeled probes and unlabeled mutant probes were incubated with the reaction mixture prior to adding the labeled probe.

### Chromatin Immunoprecipitation (ChIP) Assay

1×10^7^ cells were cross-linked with 1% formaldehyde for 10 min at 37°C. Then cells were washed with cold PBS, harvested by scraping and resuspended in cell lysis buffer containing protease inhibitors (Roche, USA). After incubation for 10 min on ice, cells were sonicated to generate about 200–1000 bp DNA fragments, and centrifuged for 10 min at 4°C. The supernatants were divided and incubated with either anti-Ets-2 antibody (Santa Cruz, USA), or IgG (Boster, China) as a negative control at 4°C overnight with rotation, respectively. The immune complexes were precipitated with salmon sperm DNA/protein G-agarose (Millipore, USA) for 2 h at 4°C, then were recovered, washed, eluted with the ChIP elution buffer and reverse cross-linked by heating at 65°C for 4 h. DNA was purified from immunoprecipitation with antibody or from input samples and were analyzed by PCR, using primers flanking Ets-2 binding site in UCA1 gene promoter (Table 1). ChIP assays were performed in duplicates.

### Western Blot

Cells were pelleted and then lysed by RIPA buffer (Thermo Scientific, USA). After SDS-PAGE resolution and membrane transfer, the target proteins were probed with antibody against human Ets-2 (Santa Cruz, USA), Akt, pAkt (Cell Signaling, USA), Bax (Epitomics, USA) or GAPDH (Abmart, China) followed by incubation with horseradish peroxidase-conjugated secondary immunoglobulin antibodies (Santa Cruz, USA). Finally, the bands were visualized by chemiluminescence using a chemiluminescence detection kit (Pierce, USA) and the specific bands were recorded on X-ray film.

### Apoptosis Assay

To quantify apoptosis, cells were stained with Annexin V and PI using Annexin V-FITC/PI Apoptosis detection kit (Keygene Biotech, China) following the manufacturer instructions. Fluorescence was detected by fluorescence microscope (Olympus, Japan) and flow cytometry CyAn ADP 9 color from Beckman Coulter (Beckman Coulter Brea, CA). Flow cytometry experiments were performed in triplicates and repeated three times.

### Statistical Analysis

Data were presented as the means ± standard deviation (SD) from three separate experiments. Statistical analyses were performed using SPSS 13.0 software. Differences between groups were analyzed using the Student *t*-test. *P*<0.05 value was considered as statistically significant.

## Results

### Cloning and Bioinformatial Analysis of the UCA1 Promoter Region

**Figure 1:**
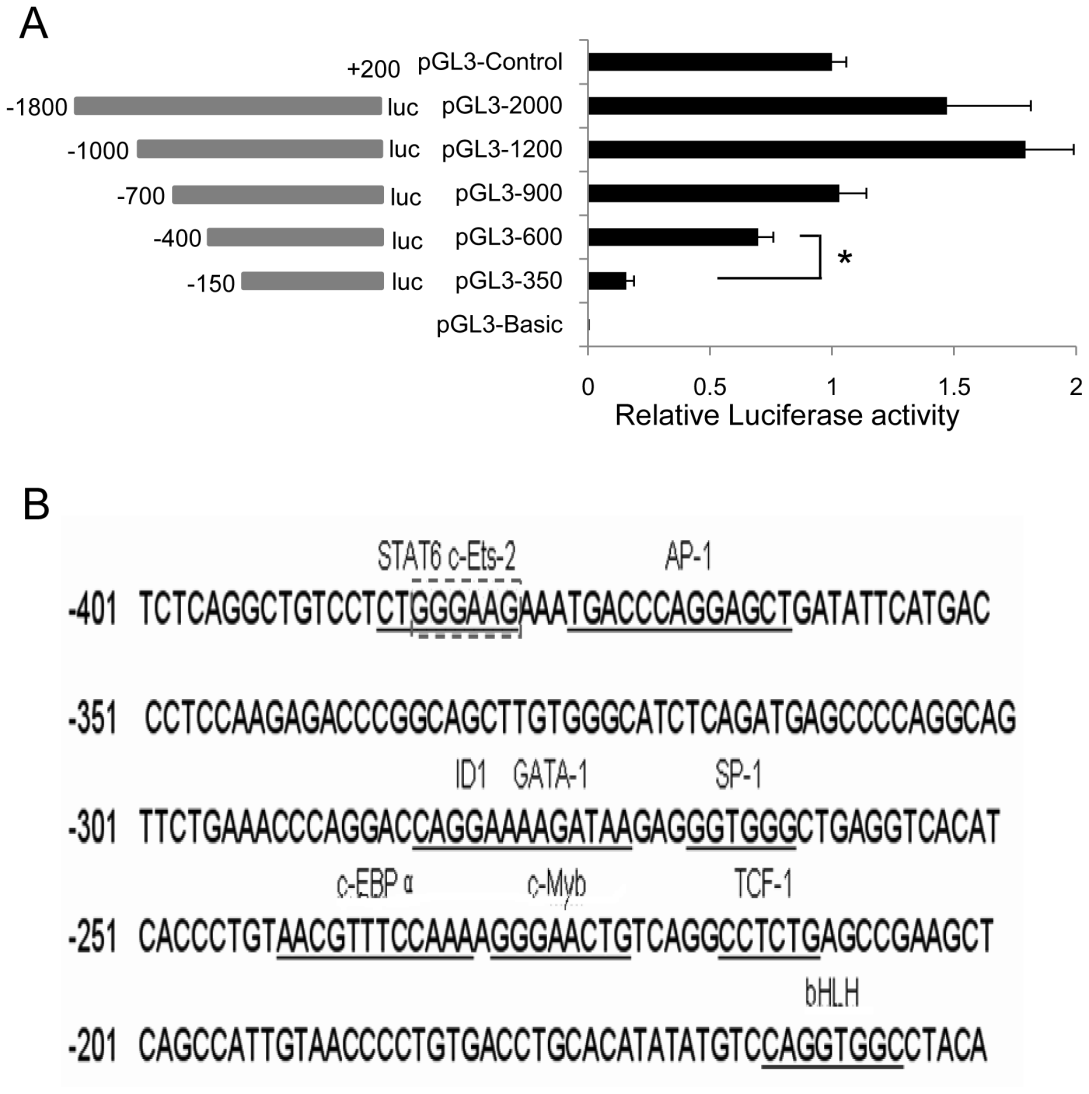
Determining the core promoter region of the UCA1 gene and its putative transcriptional factors by bioinformatical prediction. (A) Luciferase assay. Five truncated DNA fragments of the UCA1 promoter region in pGL3 vectors, a negative control vector, pGL3-basic, and positive control vector, pGL3-control, were transfected into BLZ-211 cells. Luciferase activities were measured at 48 h after transfection. Values represent means ± SD of at least three independent experiments. *P<0.05. (B) Transcriptional factors prediction by using MatInspector software. Transcription factors with high prediction score were present.

In previous study, the transcription start site (TSS) of UCA1 was identified by 5′-RACE [12]. The MegaBLAST program of NCBI was utilized to determine the TSS of UCA1 gene, which was located at 15939757 position of chr19 (GRCh37/hg19). Marking this site as +1, a total 3.0 kb DNA sequences of the UCA1 5′ flanking regulation region (−2000 bp–+1000 bp) was obtained from GenBank, which was used for bioinformatical analysis. The result of FPROM program suggested that the TSS is located at +135 bp, and a TATA-box at +105 bp. The analysis of PromoterInspector program indicated that the promoter region is between −515 bp and +100 bp region. Considering the previous 5′-RACE results and the prediction results, we chose the sequences from −1800 bp to +200 bp for following research. To evaluate the UCA1 promoter activity, reporter constructs containing a series of the 5′-flanking sequences (pGL3-UCA1-2000, pGL3-UCA1-1200, pGL3-UCA1-900, pGL3-UCA1-600, pGL3-UCA1-350) were measured by luciferase assay in BLZ-211 cells. The constructs pGL3-UCA1-2000, pGL3-UCA1-1200, pGL3-UCA1-900 and pGL3-UCA1-600 displayed different promoter activity. The pGL3-UAC1-1200 yielded the strongest promoter activity in these constructs. Notably, when the DNA fragment between −400 bp and −150 bp was deleted, the promoter activity was almost abolished (Fig. 1A), suggesting the existence of a critical positive regulatory element in this region. These deletion experiments suggest that the UCA1 promoter activity detected in BLZ-211 cells is mostly attributable to the positive regulatory element between −400 bp and −150 bp of the promoter region. There were many transcription factor (TF)-binding sites in this region predicted by the bioinformatics program MatInspector, including putative Ets-2, C/EBP, SP-1, c-Myb, *et, al*. (Fig. 1B). From these transcription factors, we chose Ets-2 as the potential TF due to its highest prediction score.

### Ets-2-binding Sites Contribute to the Promoter Activation

**Figure 2:**
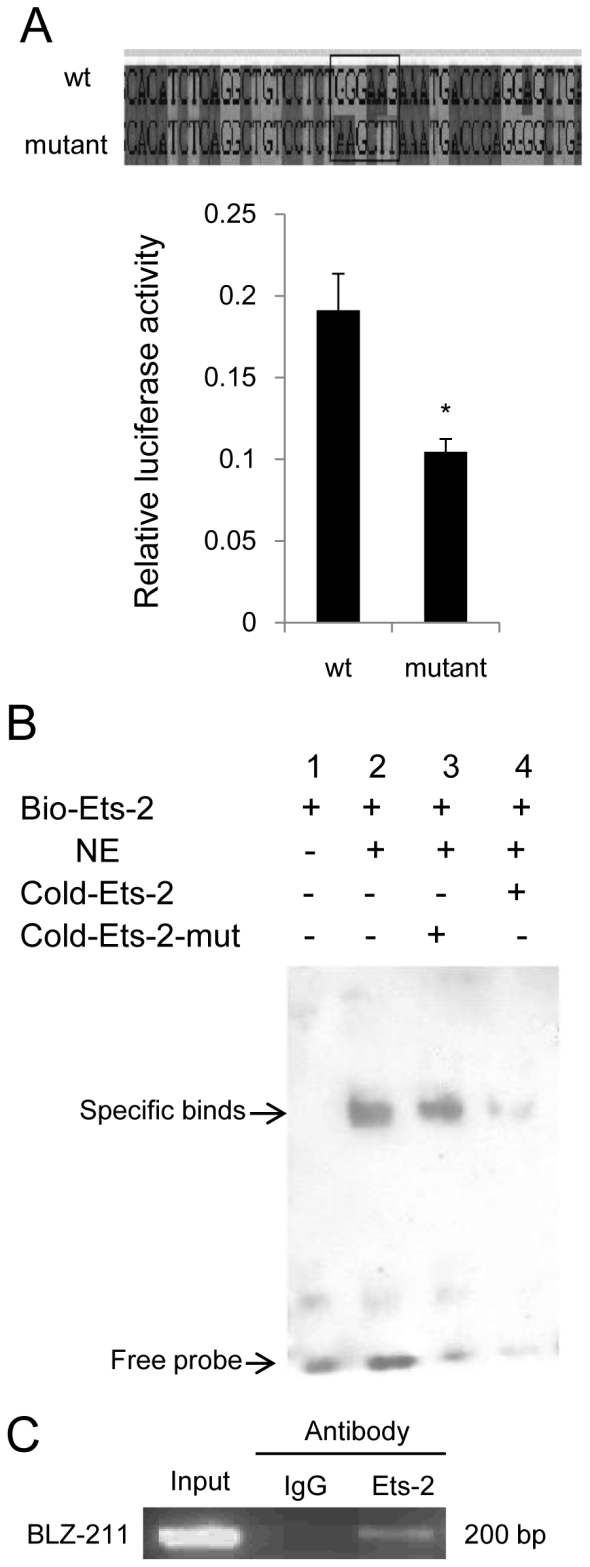
Ets-2-binding sites contribute to promoter activation of the UCA1. (A) Luciferase assay. The Ets-2 binding sites (GGGAAG) were replaced with *Hind*III sites (AAGCTT) by site-directed mutagenesis (upper panel). Relative luciferase activities of the wild-type (wt) and the mutant vectors (mutant) were measured by luciferase assay. Values represent means ± SD of at least three independent experiments. *P<0.05. (B) *In vitro* detection of Ets-2 transcription factor binding to the promoter region of the UCA1 gene by EMSA assay. One predominant band was observed when biotin-labeled Ets-2 probe was incubated with the nuclear extract (lane 2). Ets-2 binding could not be inhibited by the mutant probe (lane 3) while it was competed with the unlabeled cold probe (lane 4). (C) *In vivo* detection of Ets-2 transcription factor binding to the promoter region of the UCA1 gene promoter by ChIP assay. Input chromatin (Input), which represented portions of sonicated chromatin before immunoprecipitation, was used as a positive control. IgG antibody was used as a negtive control of ChIP assay.

To investigate whether Ets-2 is critical for UCA1 promoter activity, we replaced the Ets-2 binding sites to the *Hind*III restriction endonuclease sites in pGL3-UCA1-1200-mut. Compared to the reporter luciferase activity of wild type pGL3-UCA1-1200, the pGL3-UCA1-1200-mut yielded a much lower promoter activity in BLZ-211 cells (Fig. 2A), demonstrating that these Ets-2 binding sites indeed played an essential role in the regulation of the UCA1 promoter activity. To confirm if transcription factor Ets-2 can bind to this region, nuclear extract from BLZ-211 cells was prepared and EMSA was performed. The 22 bp oligonucleotides from −378 to −357 nt containing the Ets-2 binding sites was used as the wild type probe and the same region with mutated binding sites was used as the mutant probe (Fig. 2B). As shown in Fig. 2B, one prominent DNA–protein complex was formed with the wild-type probe. The binding was competed off by a 200-fold excess of unlabeled wild type probe, but not by a 200-fold excess of unlabeled mutant probe. The results indicated that this region was responsible for the DNA-protein complex formation. To further investigate whether Ets-2 can bind directly to the proximal UCA1 promoter *in vivo*, ChIP was performed using antibody against Ets-2 to immunoprecipitate formaldehyde-fixed chromatin from BLZ-211 cells. As shown in Fig. 2C, prominent PCR products containing the Ets-2 binding sites were detected using DNA pulled down by antibody against Ets-2, whereas no amplification was observed in the DNA immunoprecipitated by IgG, demonstrating that Ets-2 can directly bind to the UCA1 promoter in BLZ-211 cells.

### Ets-2 is Essential to the Transcription Regulation of UCA1

**Figure 3:**
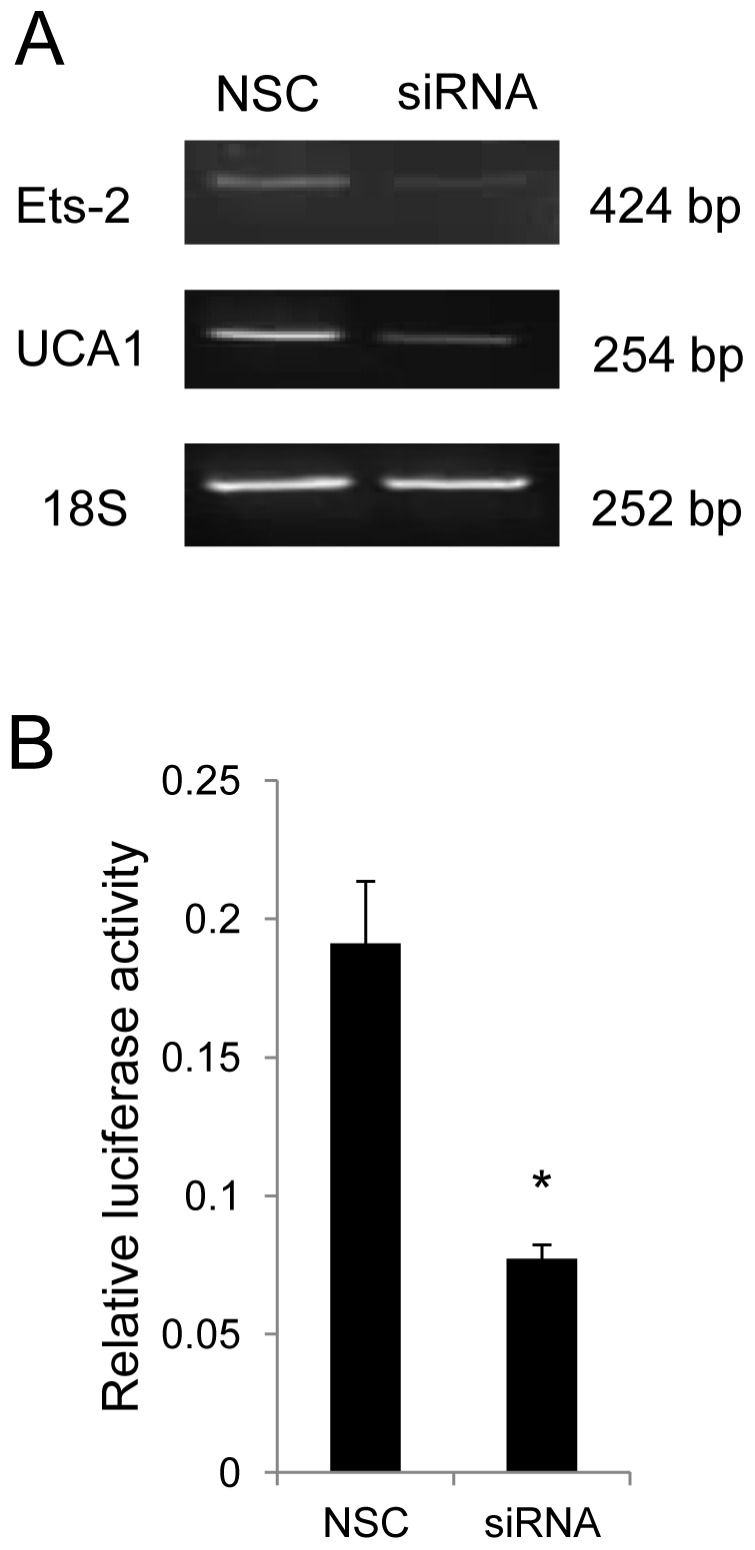
Ets-2 is essential to the transcription regulation of the UCA1. (A) Expression of Ets-2 and UCA1 was measured by RT-PCR following Ets-2 specific siRNA or scrambled siRNA treatment in BLZ-211 cells. 18 S rRNA was used as an internal control. (B) Luciferase assay. Ets-2 specific siRNA or scrambled control siRNA was co-transfected with pGL3-UCA1-1200 into BLZ-211 cells. Values represent means ± SD of at least three independent experiments. *P<0.05. NSC: non-specific control siRNA.

To determine whether transcription factor Ets-2 could regulate UCA1 expression, semi-quantitive RT-PCR was performed after knocking down Ets-2 by specific siRNA in BLZ-211 cells. The PCR results showed that the UCA1 mRNA expression was decreased by around 50% following the attenuation of the Ets-2 expression (Fig. 3A). Furthermore, as the luciferase assay results shown, when we co-transfected the Ets-2 specific siRNA or scrambled control siRNA with pGL3-UCA1-1200 into BLZ-211 cells, knockdown of Ets-2 caused the decreased activity of UCA1 promoter in comparison with the scrambled control siRNA group (Fig. 3B). These results indicated that Ets-2 can regulate the expression of UCA1 by affecting the activity of the UCA1 promoter.

### UCA1 may be Involved in the Apoptosis Induced by Ets-2 Knockdown in BLZ-211 Cells

**Figure 4:**
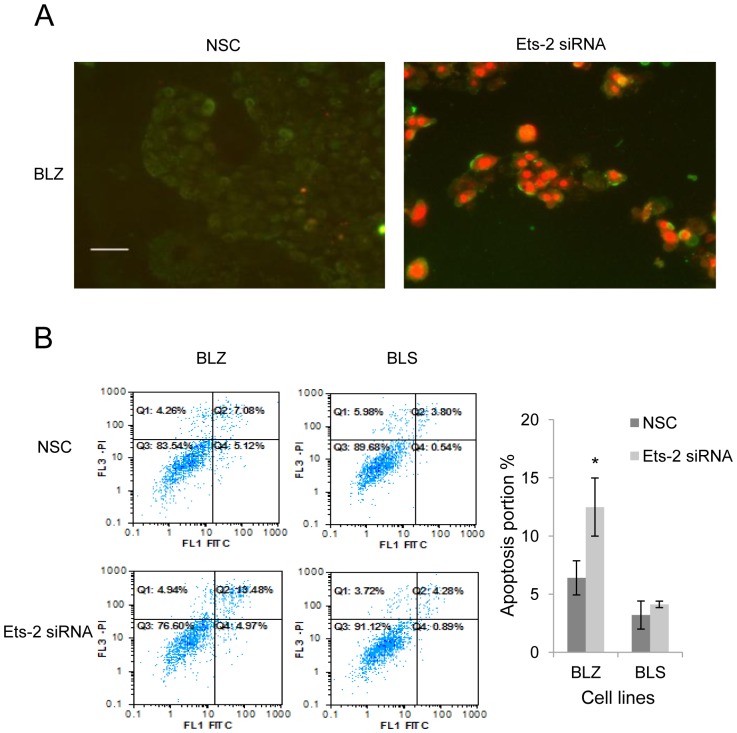
UCA1 may be involved in apoptosis induced by Ets-2 knockdown in BLZ-211 cells. (A) The induction of apoptosis following a 48-hour treatment of Ets-2 siRNA or scrambled control siRNAs in BLZ-211 cells was examined by using a fluorescence microscope. Cells were stained with Annexin V (Green) and PI (Red). Scale bar 25 µm. The images were representative of three independent experiments. (B) Apoptosis was quantified by flow cytometry (FCM). The FCM results were representative of three independent experiments. Cellular pro-apoptosis processes (upper right quadrant) were increased after Ets-2 knockdown in BLZ-211 cells (12.49±1.21% vs. 6.40±1.47%, *P<0.05) while no significant change in BLS-211 cells (4.12±0.28% vs. 3.20±2.50%, *P*>0.05). NSC: non-specific control siRNA.

The role of Ets-2 in bladder cancer is unclear, thus we verified the functional consequence of Ets-2 knockdown in bladder cancer cells. An increase level of apoptosis was observed following a 48-hour treatment with Ets-2 siRNAs in BLZ-211 cells via fluorescence microscope. The results of cell apoptosis assays showed an increased level of apoptosis in BLZ-211 cells when cells were treated with Ets-2 specific siRNA compared to the scrambled control siRNA treatment (Fig. 4A). To verify if Ets-2 induced UCA1 gene expression was involved in apoptosis we studied another bladder TCC cell line, called BLS-211. BLS-211 is lack of endogenous UCA1 expression and is derived from the same patient as BLZ-211 cell line. Interestingly, after knocking down the Ets-2 gene, we didn’t observed subsequent apoptosis in BLS-211 cells (Fig. S1). Additionally, we counted the apoptotic cells using flow cytometry in both cell lines separately. Consistent with the fluorescence microscope results, the flow cytometry results revealed a significant increase of cell pro-apoptosis (upper right quadrant) after Ets-2 knockdown in BLZ-211 cells (12.49±1.21% *vs.* 6.40±1.47%) while no increase of pro-apoptosis was observed after Ets-2 knockdown in BLS-211 cells (4.12±0.28% *vs.* 3.20±2.50%) (Fig. 4B). These results suggested that UCA1 may be involved in the cell apoptosis induced by the suppression of the Ets-2.

### UCA1 may Participate in the Inactivation of Akt Pathway in the Cell Apoptosis Induced by Ets-2 Down-regulation

**Figure 5:**
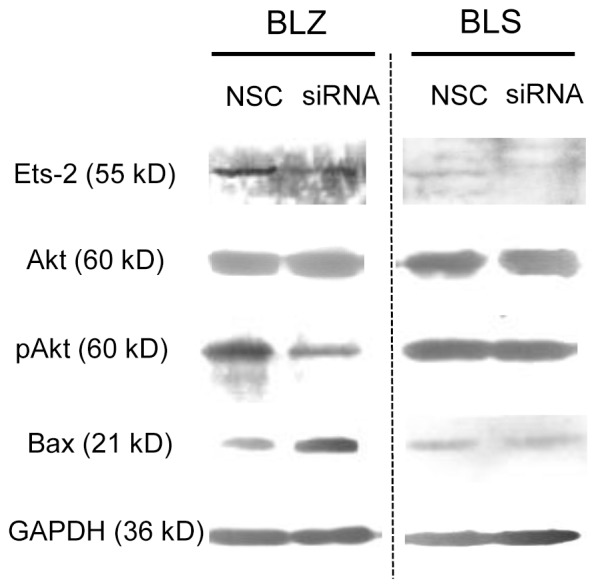
UCA1 may participate in the inactivation of Akt apoptotic pathway induced by Ets-2 down-regulation. Ets-2 and apoptosis related proteins were detected by Western blot. GAPDH was used as an internal control. NSC: non-specific control siRNA.

**Figure 6:**
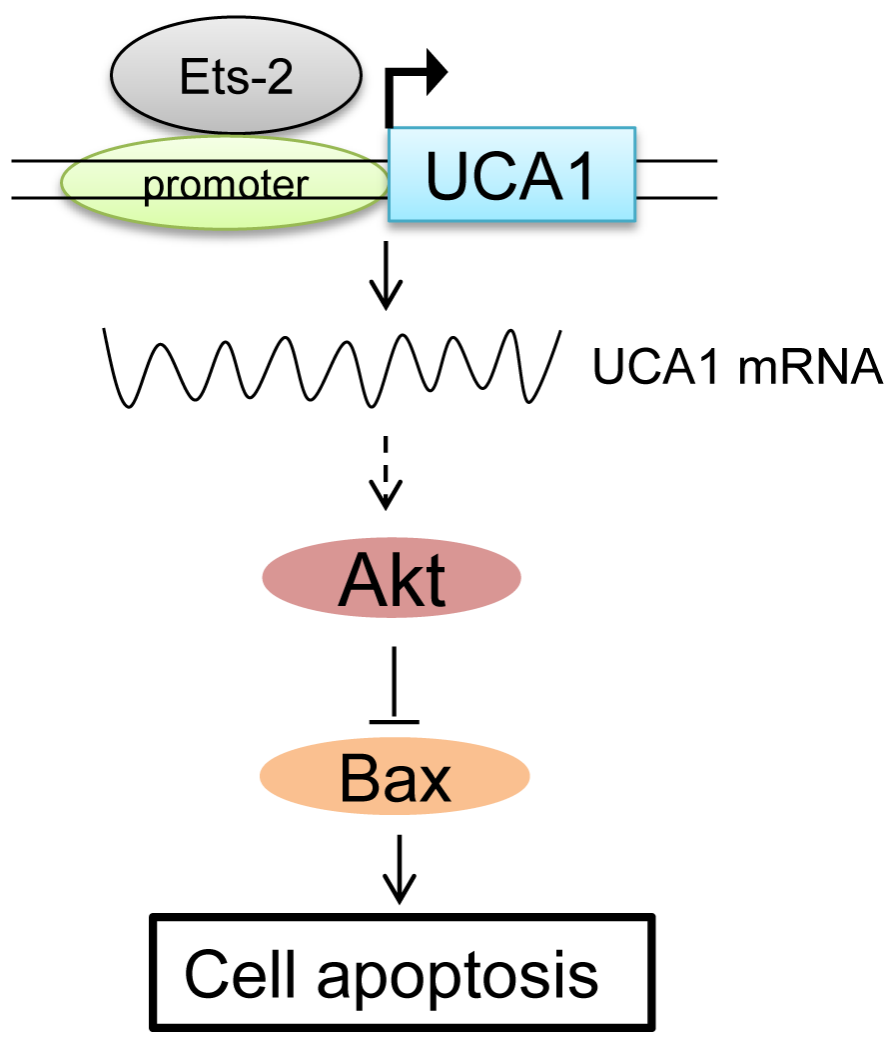
Schematic diagram of UCA1 regulation by Ets-2. Dashed line from UCA1 to Akt indicates that the association between UCA1 and Akt may be indirect.

To determine whether suppression of Ets-2 induce apoptosis in bladder cancer cells via Akt pathway, a privotal apoptosis regulator pathway, we investigated the protein expression level of total Akt and phosphorylated Akt (pAkt). Interestingly, the western blotting results showed that after silencing the Ets-2, pAkt expression was subsequently decreased, while expression of proapoptotic protein Bax was increased (Fig. 5, left panel). Our results implied that Ets-2 knockdown enabled to induce apoptosis in BLZ-211 cells via inactivation of Akt pathway and led to changes in the downstream target molecules. Thus we assumed that UCA1 gene expression may be associated with the activation of Akt pathway which is involved in the Ets-2 mediated anti-apoptosis process in bladder cancer cells. To address this concept, we next studied the protein expression level of Akt, pAkt and Bax in BLS-211 cells. As shown, it was no noticeable difference between Ets-2 siRNA treated group and scrambled siRNA treated group (Fig. 5, right panel). Our results suggested that down-regulation of Ets-2, a transcription factor of the UCA1 gene, can induce apoptosis in BLZ-211 cells by decreasing the UCA1 expression and this may contribute to the inactivation of the Akt pathway (Fig. 6).

## Discussion

Bladder cancer is one of the most common malignancies in China, ranking as the first frequent neoplasm of the urinary tract. We have previously demonstrated that UCA1 can promote cell growth and invasion in bladder cancer [12]. UCA1 is a lncRNA, since it has multiple small ORF though no strong Kozak consensus sequences were found in any of the ATG codons [12], [13]. Several lncRNAs have been identified that are linked to human diseases and have specific functions related to human diseases [19]. Previously, we demonstrated the spatial and temporal expression pattern of UCA1. However, the transcriptional regulation of UCA1 gene expression has not been studied. Here, for the first time we have characterized the promoter region of the human UCA1 gene and reported its regulation by Ets-2 transcription factor.

In contrast to conventional protein-coding genes, the sequences, the transcriptional start sites, the exon structures, and the lengths of these long non-coding genes are all highly variable [20]. Based on their genomic proximity to protein-coding genes, lncRNAs are classified as overlapping, cis-antisense, bidirectional or intronic ncRNAs [21], [22].The mechanisms of lncRNA expression are unknown, although there is much evidence to suggest that lncRNAs are regulated by similar mechanisms as protein coding genes. Briefly, in the study of Wang et al, they found a core promoter region (from nucleotides −132 to −48) of the lncRNA HULC gene and they characterized a CREB binding site between nucleotides −67 and −53 in the core promoter region [23]. In another study by Zhao et al, they analyzed a 5 kb genomic DNA fragment upstream of the first exon of the lncRNA MEG3 gene, characterized a CRE site involved in MEG3 gene transcription and also found several proteins from the CREB family directly bind to the CRE site [24]. Moreover, many studies suggest that the mechanisms by which ncRNA expression is altered in cancers are similar to those for protein coding genes, including epigenetic mechanisms [25], [26], [27], [28].

In our study, we analyzed the proximal promoter region of the UCA1 which located at −2000 nucleotides upstream of transcription start site of the UCA1 gene. We demonstrated by luciferase activity assay and deletion analysis that the region between nucleotides −400 to −150 may contribute to the basic promoter activity. And in this region we predicted many known transcription factor via bioinformatics prediction methods. We also demonstrated by EMSA and ChIP analysis that the Ets-2 binding sites between nucleotides −385 and −380 played an important role in the regulation of the UCA1 promoter activity. In our study we used BLS-211 cells, which were failed to express the UCA1 gene although they expressed the Ets-2 gene. When we treated the cells with Ets-2 siRNA, the UCA1 promoter activity was not completely abolished. Both observations suggest that UCA1 expression may be controlled by other transcriptional factors as well. Noteworthy when the region between nucleotides −700 and −400 was deleted, the promoter activity considerably decreased. It suggests a presence of some transcriptional activators in this region. For further clarification future studies need to be focused on other TFs of the promoter region of the UCA1 gene, which contribute to the activity of the UCA1 gene.

Ets-2 was initially characterized as a proto-oncogene. Over the years the Ets transcription factor family became a common element in tumourigenesis [29]. Findings by Carbone *et al* suggested that downregulation of Ets-2 in prostate cancer cells was associated with reduced levels of the anti-apoptotic protein bcl-x(L), growth regulatory factors cyclin D1, and c-myc. This study revealed the specific role of Ets-2 in promoting growth and survival of prostate cancer cells [30]. In another study, they showed that transient expression of Ets-2 protect cells from apoptosis by increasing the promoter activity of Bcl-xl and consequently enhancing its mRNA and protein expression levels in mesothelioma cell lines [31]. Previously, Hanke *et al* found that the ratio of Ets-2 mRNA to urokinase plasminogen activator (uPA) mRNA in urine could be a potential marker for bladder cancer [32]. Although there are data supported that Ets-2 takes part in bladder cancer development, the exact roles and mechanisms of Ets TFs in bladder cancer are mostly unknown.

In our study, we showed an inhibition of UCA1 expression induced by Ets-2 gene silencing. Functional consequence of Ets-2 gene silencing led to apoptosis in BLZ-211 cells. We also demonstrated that Akt pathway was involved in this process. The serine–threonine kinase Akt (also known as protein kinase B) is a central convergence node in a broadly influential signaling network. Akt regulates essential cellular functions such as migration, proliferation, differentiation, apoptosis, and metabolism [33]. In addition, activation of Akt pathway plays a pivotal role in the inhibition of apoptosis induced by a number of stimuli including growth factor withdrawal, detachment of extracellular matrix, UV irradiation, cell cycle discordance and activation of FAS signaling [34], [35], [36]. To test whether Ets-2 regulates Akt pathway through UCA1, we used another bladder cancer cell line BLS-211, which lacks of UCA1 gene expression and it is derived from the same patient as BLZ-211. Most interestingly, following the attenuated expression of Ets-2 in BLS-211 cells we did not observe down-regulation of pAkt and increase of cell apoptosis. These results showed that UCA1 may be involved in the activation of pAkt pathway by Ets-2 (Fig. 6). These results were consistent with our previous study showed that pAkt is down-regulated in the UCA1 stable knockdown BLZ-211 cells [37]. LncRNAs have been shown to harbor biological activities. So far as is known, the broad functional repertoire of lncRNAs includes roles in high-order chromosomal dynamics, telomere biology and subcellular structural organization [22], [38]. Besides our study, other studies also suggested that lncRNA may be involved in the regulation of signal pathway, such as wnt pathway, Ras pathway [39], [40]. However, the mechanism that how Ets-2 regulates Akt pathway remains unclear and whether the Akt signaling proteins directly interact with UCA1 needs to be proved further.

In summary, we looked at regulatory elements directly involved in UCA1 gene transcription and found that the promoter activity of UCA1 is mainly attributable to the Ets-2 binding site within the proximal promoter region. We demonstrated that transcription factor Ets-2 can directly bind to this site and it is essential to the transcriptional control of UCA1 expression. In addition, our results revealed that UCA1 may be involved in the regulation of Akt pathway by Ets-2. Therefore, we concluded that Ets-2 regulates the expression of UCA1, moreover lncRNA UCA1 may be involved in the activation of Akt signaling pathway by Ets-2. Our findings advocate for further research on this field. This is a novel field with continuously increasing importance. It is a rising importance and interest to explore the function and regulation of these lncRNAs.

## Supporting Information

Figure S1 Knockdown of Ets-2 cannot induce apoptosis in BLS-211 cells. The induction of apoptosis following a 48-hour treatment of Ets-2 siRNA or scrambled control siRNAs in BLS-211 cells was examined by using a fluorescence microscope. Cells were stained with Annexin V (Green) and PI (Red).
(TIF)
