# Supplementary figures and images for: Ets-2 Regulates Cell Apoptosis via the Akt Pathway, through the Regulation of Urothelial Cancer Associated 1, a Long Non-Coding RNA, in Bladder Cancer Cells
Source: PLoS One. 2013 Sep 12;8(9):e73920. doi: 10.1371/journal.pone.0073920 (PMC3771932; doi:10.1371/journal.pone.0073920)

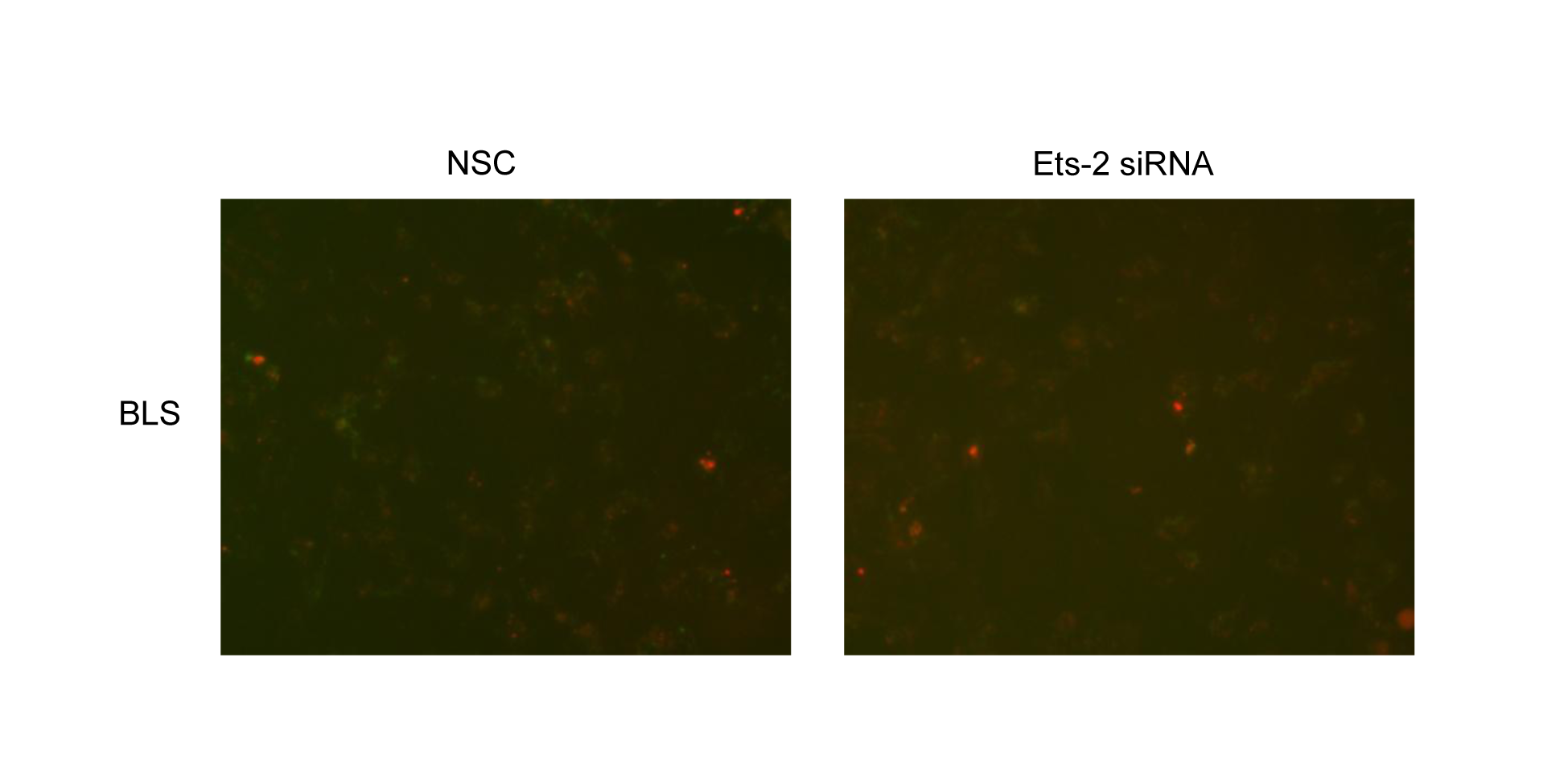

Supplement: Figure S1 — Knockdown of Ets-2 cannot induce apoptosis in BLS-211 cells. The induction of apoptosis following a 48-hour treatment of Ets-2 siRNA or scrambled control siRNAs in BLS-211 cells was examined by using a fluorescence microscope. Cells were stained with Annexin V (Green) and PI (Red). (TIF) [file pone.0073920.s001.tif]
